# Forthcoming Developments Indicated in the Budget

**Published:** 1914-05-23

**Authors:** 


### NATIONAL INSURANCE ACT DEVELOPMENTS.

### Forthcoming Developments Indicated in the Budget.

Some far-reaching developments of the present plan of National Insurance were indicated by the Chancellor of the Exchequer in his recent Budget speech. The term " indicated " is used advisedly, for the barest outline of the proposed alterations was all that was given. The extra cost to the State involved by the proposals is, however, estimated at no less than £1,250,000 per annum, so that it is clear that some substantial alterations are in contemplation. In spite of the very meagre particulars that were given it is possible to arrive at certain conclusions as to the intentions of the Government, and it is thought that an attempt to outline the difficulties that will have to be overcome in carrying out the suggested alterations will not be without interest.

Fresh legislation will be required to give effect to the proposals, and this will necessarily imply the introduction of another National Insurance Act Amendment Bill during the present session of Parliament, and as some of the alterations indicated are of a contentious character it is clear that early consideration of the principles involved is of the utmost importance.

### The Finances of Approved Societies.

The first amendment indicated by the Chancellor referred to the financial position of certain approved societies, and he announced the intention of the Government to come to their assistance. On the whole, we were told that in spite of the abnormal work of the first year the resources provided fully justified the actuarial estimate. Experience has shown, however, that certain well-managed societies have attracted an unusual proportion of " bad " lives—in the insurance meaning of that term—or were limited to persons subject to special sickness or disability. It has also been made clear that there is an appalling amount of sickness among married women under certain conditions—a fact that was quite unknown before the Act came into operation. These considerations have led the Government to the conclusion that some assistance must be given to well-managed societies, where deficiencies are shown to exist which are not due to malingering and which are beyond the control of the societies concerned. The conditions in some cases are said to be very abnormal, and it is known that some societies have already actually exceeded their credit with the Insurance Commissioners and have had to draw on their reserves. The Chancellor described them as distressing cases.

But while promising State assistance the Chancellor indicated that the whole of the burden will not fall upon the State, and that it will be necessary to call upon the societies to come to the rescue. This is an admission that the fundamental basis of the administration of the Act through independent approved societies, which are allowed to select their members as they choose, is unsatisfactory in its practical application, but it is by no means clear how the proposed assistance can be given without a fundamental alteration of the present system.

In the first place, it is very easy to say that assistance shall only be given to societies when the deficiencies are due to causes that are beyond their control, and that no assistance shall be given when they arise from mismanagement or malingering, but it will be a very difficult matter in practice to determine when the respective conditions prevail. Few societies, if any, as at present constituted, would agree to accept the charge of mismanagement, or to admit that an excess of claims was due to systematic malingering, and it would be an equally difficult task to show to what extent the composition of any society is abnormal. It will, of course, be necessary to fix some standard for comparison, and effect will have to be given in this to the age distribution of the members, while it is clear that a different standard will be required for women from that applicable to men. Futhermore, it would appear that in the case of women's societies account will have to be taken of the condition of the members as to marriage, and it is not clear that a satisfactory standard for such societies is yet available.

### Will Approved Societies have to " Pool " their Funds?

In the second place it would seem from the statement of the Chancellor that " the whole of the burden will not fall upon the State, and the societies must come to the rescue," that some scheme for " pooling " surpluses and deficiencies is in contemplation. Now it is laid down in the principal Act that those societies which have a membership of at least five thousand will stand by themselves for purposes of valuation and in the distribution of surplus. This provision was intended to give a stimulus to careful management, and many societies were formed with the specific purpose of securing to their members the advantages to be gained by the light rate of sickness that was anticipated in the class from which its members were to be drawn, and to give to such members the additional benefits derived from the light sickness experience, coupled with the fruits of good management. It is hardly likely, in the circumstances, that the executives of these societies will willingly agree to the proposal for pooling funds with societies which admittedly experience heavy sickness rates, and, in fact, it may be anticipated that strenuous resistance will be made against any such proposition.

If, indeed, the proposal be seriously made it would be to the interest of these societies to relax the strict supervision of claims and to cease working for a surplus, and we have little doubt that the self-interest would in some way predominate, so that the proposal would defeat its own ends by increasing the claims on societies whose sickness rates are now low, without any compensating advantage to societies whose sickness claims are high. It is little wonder, therefore, that the Chancellor indicated that it would not be desirable to go into details of the proposal in the course of his Budget speech, and that it would be necessary to consult with the whole of the societies, and particularly with those immediately concerned, before finally deciding how the extra State assistance is to be bestowed.

It may also be pointed out that the idea of separate societies of more than five thousand members pooling their surpluses and deficiencies is contrary to the spirit of the Act in another way, for particular care has been taken—principally at the instigation of Scotland—that in the case of a society which has members in more than one part of the United Kingdom it is to treat its members in each country as a separate society, for the purpose in question, should the members in either country so desire.

### Are the Rates of Benefit Guaranteed?

It is thus apparent that there are many pitfalls to be avoided in the distribution of the extra State grant to approved societies. Even without requiring the societies themselves to come to the rescue it is difficult to see, when once the principle of differentiation is admitted, that the State can do otherwise than guarantee the payment of the benefits at the rates set forth in the Acts. The ostensible purpose of the extra grant is to enable those benefits to be given by the societies, even though a deficiency be shown on an actuarial valuation, and thus to avoid the necessity of reducing the benefits or increasing the contributions, both of which courses would be unpopular. So far no such guarantee has been given, and any steps that may be taken in that direction must be scrutinised with care in order that their true import upon the future cost to the State of the National Insurance scheme may be fully appreciated. No inconsiderable proportion of the excess sickness claims is due to the overlapping of the State insurance with previously existing voluntary insurance through the friendly societies. Insurance, as a means of co-operation, is a healthy growth of social legislation, but over-insurance is a social menace; and it is to this that the attention of the Government should be largely directed.

This leads us to the principal point here emphasised—namely, that the Government's legislative proposals for giving effect to the suggested alterations should be brought forward at the earliest possible moment, in order that they may be fully and impartially considered both in and out of the House.

### Deposit Contributors.

The hard case of a certain proportion of the deposit contributors is also indicated as a matter for further State assistance. Here, again, to judge from the statements of the Chancellor, it is intended to exercise some discrimination as to the apportionment of the grant, for he made it clear that many deposit contributors were such from choice and not of necessity. We dealt with the possible consequences of the revision of the conditions regulating deposit insurance a few weeks ago. At that time it appeared that there was some possibility of the revision being allowed to remain in abeyance this year, in spite of the requirement of the Act of 1911, and that the present conditions would be extended under the Expiring Laws Extension Act. It is satisfactory to note that this course is not to be adopted, and an opportunity has now arisen for putting the system on a satisfactory permanent basis, even though it may necessitate the formation of a State society conducted on insurance principles as contrasted with the present plan which, in reality, is not insurance at all.

### Provision against Serious Illness.

Until details are furnished it would be captious to criticise the proposal to establish clinics in convenient centres to assist the panel doctors to cooperate in difficult cases. Nor is there any reason to doubt the advantage that will accrue from the proposal, if carried out successfully, to establish a system of medical referees who will not merely be a check upon malingering, but will be expert medical men who can be called in for an opinion where there is a serious case of illness, and the ordinary medical man on the panel is not quite sure and wants to get a second opinion. These were amendments strongly advocated by the Fabian Committee, whose report was recently considered somewhat fully in our pages, and provided the grant that is made is sufficient to secure a satisfactory service, one of the principal causes for dissatisfaction with the Act—namely, its lack of provision for serious illness—will be eliminated.

Altogether the new proposals are exceedingly interesting, and we shall watch developments during the course of the next few weeks as the intentions of the Government are more fully expounded.

